# *In Silico* Identification of Structure Requirement for Novel Thiazole and Oxazole Derivatives as Potent Fructose 1,6-Bisphosphatase Inhibitors

**DOI:** 10.3390/ijms12118161

**Published:** 2011-11-18

**Authors:** Ming Hao, Xiaole Zhang, Hong Ren, Yan Li, Shuwei Zhang, Fang Luo, Mingjuan Ji, Guohui Li, Ling Yang

**Affiliations:** 1 Department of Materials Science and Chemical Engineering, Dalian University of Technology, Dalian, Liaoning, 116023, China; E-Mails: dluthm@yeah.net (M.H.); zswei@dlut.edu.cn (S.Z.); 2 Department of Mathematical Sciences, Dalian University of Technology, Dalian, Liaoning, 116023, China; E-Mail: xlfree@foxmail.com; 3 Department of Ophthalmology, Qi Lu Hospital, Medical School of Shandong University, Jinan, 250012, China; E-Mail: renhong999@sina.com; 4 Laboratory of Molecular Modeling and Design, State Key Laboratory of Molecular Reaction Dynamics, Dalian Institute of Chemical Physics, Chinese Academy of Sciences, Dalian, 116023, China; E-Mail: ghli@dicp.ac.cn; 5 College of Chemistry and Chemical Engineering, Graduate School of the Chinese Academy of Sciences, Beijing, 100049, China; E-Mails: luofang09b@mails.gucas.ac.cn (F.L.); jmj@gucas.ac.cn (M.J.); 6 Laboratory of Pharmaceutical Resource Discovery, Dalian Institute of Chemical Physics, Graduate School of the Chinese Academy of Sciences, Dalian, Liaoning, 116023, China; E-Mail: yling@dicp.ac.cn

**Keywords:** 3D-QSAR, molecular dynamics, FBPase inhibitors, CoMFA, CoMSIA

## Abstract

Fructose 1,6-bisphosphatase (FBPase) has been identified as a drug discovery target for lowering glucose in type 2 diabetes mellitus. In this study, a large series of 105 FBPase inhibitors were studied using a combinational method by 3D-QSAR, molecular docking and molecular dynamics simulations for a further improvement in potency. The optimal 3D models exhibit high statistical significance of the results, especially for the CoMFA results with *r*_ncv_^2^, *q*^2^ values of 0.986, 0.514 for internal validation, and *r*_pred_^2^, *r*_m_^2^ statistics of 0.902, 0.828 statistics for external validation. Graphic representation of the results, as contoured 3D coefficient plots, also provides a clue to the reasonable modification of molecules. (1) Substituents with a proper length and size at the C5 position of the thiazole core are required to enhance the potency; (2) A small and electron-withdrawing group at the C2 position linked to the thiazole core is likely to help increase the FBPase inhibition; (3) Substituent groups as hydrogen bond acceptors at the C2 position of the furan ring are favored. In addition, the agreement between 3D-QSAR, molecular docking and molecular dynamics simulation proves the rationality of the developed models. These results, we hope, may be helpful in designing novel and potential FBPase inhibitors.

## 1. Introduction

Type 2 diabetes mellitus (T2DM) is a chronic metabolic disorder that results from defects in both insulin secretion and insulin action. Nowadays, T2DM, as a major health hazard, is widespread and sometimes even fatal, which leads to both medical and social challenges. It has been reported [1] that type 2 diabetes mellitus is a progressive disorder. Often it can be treated initially with oral drug monotherapy. However, with the progress of the disease it will require the addition of other oral drugs, and in many patients the therapy of injecting insulin will be critically required to achieve targeted glycemic levels. As we know, patients with diabetes experience significant morbidity and mortality from microvascular and macrovascular complications. It has been documented that diabetes is the primary cause of blindness [2]. Therefore, patients with diabetes and corresponding complications suffer from great mental and physical pain. It is imperative for researchers to develop safe and effective drugs to prevent this kind of obstinate disease. Currently, many reports have illustrated that diet and exercise therapy plays an important role in the treatment for patients with T2DM [3,4]. However, if this therapy fails to achieve the desired level of glycemic control, pharmacologic intervention is required. Now, several classes of oral drugs are reported to have an effect on the treatment of T2DM including sulfonylureas [5], metformin [6], repaglinide [7], troglitazone [8] and so on. However, these oral agents still have some limitations. For example, sulfonylurea therapy usually is associated with weight gain, which has been implicated as a reason of secondary drug failure [3]; Metformin therapy is forbidden in patients with renal and hepatic disease, respiratory insufficiency and alcohol abuse; and troglitazone, a very expensive oral drug, often causes hypercholesterolemia which is a major risk factor for coronary artery disease. Therefore, the development of a kind of drug with more safe profiles and less side effects is a task of top priority.

Most of the work has focused on enzymes in the gluconeogenesis (GNG) pathway, because this pathway is partly responsible for the excessive glucose production in type 2 diabetes mellitus [9]. Out of these enzymes, fructose 1,6-bisphosphatase (FBPase), a highly regulated enzyme that catalyzes the second to last step in gluconeogenesis [10,11], draws the attractiveness as a drug target to treat T2DM. FBPase enables the inhibition of gluconeogenesis from all GNG substrates while avoiding direct effects on glycogenolysis, glycolysis and the tricarboxylic acid cycle. Moreover, evidence from clinical researches illustrates that FBPase inhibitors may show an adequate safety margin [11]. Thus, several classes of drugs targeting at the FBPase, recently, have been reported including MB06322 [12], anilinoquinazolines [13], benzoxazole benzenesulfonamides [14], MDL-29951 [15], AMP mimics [16–18], *etc*. However, among these agents, some series show very poor oral bioavailability (OBAV), thus, making the development of novel drugs with acceptable OBAV and more potency required.

Nowadays, *in silico* modeling approaches [19–23], as a productive and cost-effective technology in design of novel lead compounds, have been used in combination with experimental practices [24–27] to facilitate the drug discovery process. In view of this, Chen and co-workers have carried out excellent work to study the FBPase inhibitors using the *in silico* method based on 63 FBPase inhibitors [28]. In the present work, more diverse set of molecules were performed to examine if a similar level of prediction can be achieved. In addition, besides both three-dimensional quantitative structural activity relationships (3D-QSAR) and molecular docking, in this work, a molecular dynamics (MD) approaches were also performed to investigate the stability of the docking results. Thus in the present work, a total of 105 thiazoles and oxazole-based inhibitors of FBPase was collected to build 3D-QSAR models using comparative molecular field analysis (CoMFA) [29] and comparative molecular similarity indices analysis (CoMSIA) methods [30]. The reliability and robustness of the developed best models were estimated with bootstrapping analysis and *y*-randomization check. In addition, the predictive abilities of the obtained models were validated statistically with an external test set of compounds using multiple statistical criteria. In addition, a combined computational approach including the docking analysis and molecular dynamics (MD) simulation was also employed to elucidate the probable binding modes of these antagonists at the binding site of the FBPase. The good concordance between the 3D contour maps and the docking result provides our identification of several key features of the binding mechanism for these inhibitors. We hope the developed models could provide some meaningful clues in the future synthesis of highly potent and orally bioavailable FBPase inhibitors.

## 2. Results and Discussion

### 2.1. Molecular Docking

**Figure 1:**
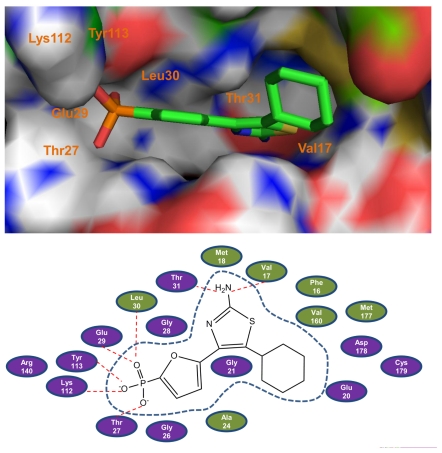
The binding models of the most potent compound **27** with Fructose 1,6-bisphosphatase (FBPase). Top panel: A surface rendering to illustrate the interactions between compound **27** with the representative key amino acids. The inhibitor is represented as stick model and carbon atoms are colored green. Bottom panel: 2D representation of compound **27** and FBPase. The active site residues are represented as follows: polar residues in purple, hydrophobic residues in olive, respectively. The red dash denotes H-bonds.

The docking results could illustrate to us the active-site architecture and ligand binding model as well as the network of electrostatic, hydrogen bond and van der Waals interactions associated with ligand binding between the inhibitors and FBPase. In this work, the most potent compound **27** is taken as the representative to elucidate this point in detail. As illustrated in Figure 1, compound **27** locates at the active site of the enzyme and binds primarily through the H-bond and van der Waals interactions. The majority of hydrogen-bond and hydrophobic modes are the same as those observed in the crystal structure of AMP complexed with FBPase [31], which validates the reliability of the docking model.

As can be seen from the figure, the phosphate of compound **27** forms hydrogen bond interactions with the main chain NH group residues of Thr27, Glu29, Leu30, with the OH of Tyr113, Thr27, and the side chain NH^3+^ group residue of Lys112. In addition, the amino group attached to the thiazole ring forms two other hydrogen bonds with the FBPase residues: one with the carbonyl O of Val17 and the other with the side chain OH of Thr31. Besides these hydrogen bond interactions, the van der Waals interactions also play a central role in the ligand binding as shown in Figure 1. The furan ring interacts with the main chain carbons of Gly26 and Gly28, with the side chain carbons of Leu30 and Val160. Interactions between the thiazole core and the binding pocket, besides hydrogen bond interactions, are mainly van der Waals contacts with the Phe16, Val17 and Met18. For the cyclohexyl ring, one can notice that the cyclohexyl ring produces van der Waals interactions with the amino residues of Ala24, Glu20, Asp178, Cys179 and Met177. All these interactions fix the inhibitor 27 in the binding pocket steadily.

Compared to the interactions between AMP and FBPase [31], the most potent compound **27** forms more van der Waals interactions with the amino residues of FBPase, which may be one possible reason exhibiting its high activity. Through the investigation of the series of thiazoles and oxazoles, an interesting common characteristic was observed for most of the inhibitors that they all have the phosphate group, as a hydrogen bond acceptor, which forms a hydrogen bond network with the amino residues of FBPase.

### 2.2. 3D-QSAR Statistical Results

To measure the predictive capability of a QSAR model, several statistical parameters including especially the cross-validated correlation coefficient (*q*^2^), non-cross-validated correlation coefficient (*r*_ncv_^2^), and standard error of estimate (*SEE*), *F*-statistic values, predicted correlation coefficient for the test set of compounds (*r*_pred_^2^) as well as the principal components (*PCs*) were calculated.

When building a 3D-QSAR model, two important elements impacting greatly on the quality of the model must be taken into account. One is the molecular alignment. In this work, both the ligand- and receptor-based alignment rules were applied, with the purpose to compare and implement the results of them for exploring the receptor-ligand interaction mechanism as really as possible. Another factor is the grid descriptors. Herein, two 3D descriptors for CoMFA (*i.e.*, the steric and electrostatic fields) and five 3D descriptors for CoMSIA (*i.e.*, the steric, electrostatic, hydrophobic, hydrogen bond (H-bond) donor and acceptor fields) as well as their 31 combinations were applied as the independent variables in the building models.

As a result, several optimal CoMFA and CoMSIA models with proper predictive performance based on the same training (77 molecules) and test set (28 molecules) were obtained, with their statistical results shown in Table 1. For the ligand-based study, the optimal CoMFA model employing both the steric and electrostatic field descriptors obtains a *LOO* cross-validated *q*^2^ of 0.514, a correlation coefficient *r*_ncv_^2^ of 0.986, a *SEE* value of 0.108 and an *F* value of 462.072 using 10 components, which indicates a good internal predictivity of the model. When being validated by the independent test set which is not included in the building of the model, an *r*_pred_^2^ = 0.902 is achieved, proving its high external predictive power. As to the field contribution, the steric and electrostatic field descriptors account for 0.563 and 0.437, respectively, to the CoMFA model.

During the ligand-based CoMSIA analysis, the model with slightly worse but still acceptable statistical performances (*q*^2^ = 0.443, *r*_ncv_^2^ = 0.874, *SEE* = 0.314, *F* = 80.809) than that of the CoMFA one was observed, with three field descriptors (steric, electrostatic, hydrogen bond acceptor) employed. The *r*_pred_^2^ value for the 28 test set compounds is 0.756. As to the relative contribution, the electrostatic field makes the greatest (0.453), followed by steric field (0.379), and the hydrogen bond acceptor field gives 0.168.

**Figure 2:**
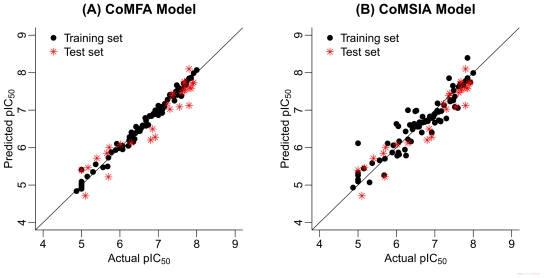
The predicted *versus* the actual pIC_50_ values for the FBPase inhibitors: (**A**) CoMFA model and (**B**) CoMSIA model.

For the receptor-based 3D-QSAR studies, it can be observed that no statistically acceptable results were obtained in terms of both internal and external validation criteria (Table 1). Thus, we focus on our further research to the optimal ligand-based CoMFA and CoMSIA models. Figure 2 illustrates the correlation plots of the experimental versus the predicted pIC_50_ values of the training (black dot) and test (red asterisk) sets for the two 3D-QSAR models. Clearly, good correlationships are observed since the predicted values are almost as accurate as the experimental activities for the whole dataset (especially for the CoMFA model). Table S1 (Supporting Information) lists the predicted results of the whole data set.

Overfitting can be a problem in QSAR. One should demonstrate that the final model is based on the correct number of components. Herein, in order to address this problem, we have validated the optimal CoMFA model using first 11 components and CoMSIA model using first 7 components. By investigating the *q*^2^, *r*_ncv_^2^ and *SEE*, we have noticed the two optimal models improve with the addition of components, that 10 components are needed for the CoMFA model and 6 for the CoMSIA model and that further improvement is not obtained with additional components (see Tables S2 and S3).

As a further test of the robustness of the CoMFA and CoMSIA models, we also randomized the target values for 50 times. As a result, none of obtained models have significant *q*^2^. The *q*^2^ values obtained are in the range from −0.630 to 0.085 for CoMFA, and from −0.476 to 0.013 for CoMSIA, respectively. This indicates that the *q*^2^ values in both the optimal CoMFA and CoMSIA models with original data are not due to chance correlations.

To testify firmly the good performance of the prediction, the squared correlation coefficient values between the actual and predicted values of the test set compounds with intercept (*r*_test_^2^) and without intercept (*r*_0_^2^) are also calculated. Table 2 gives the values of the parameters for the optimal CoMFA and CoMSIA models in the present work. According to references [32–35], models are considered acceptable if they satisfy all following conditions: (1) *r*_pred_^2^ > 0.5, (2) *r*_test_^2^ > 0.6, and (3) *r*_0_^2^ is close to *r*_test_^2^, such that the (*r*_test_^2^ − *r*_0_^2^)/*r*_test_^2^ < 0.1 and 0.85 ≤ *k* ≤ 1.15 or 0.85 ≤ *k*′ ≤ 1.15. When the observed values of the test set compounds (*X* axis) are plotted against the predicted values of the compounds (*Y* axis) setting intercept to zero, the slope of the fitted line gives the value of *k*. Interchange of the axes gives the value of *k*′. As can be seen from the table, both the best CoMFA and CoMSIA models successfully pass those tests.

A previous report [36] has illustrated that the *r*_pred_^2^ may not truly reflect the predictive capability of a model on a new dataset. Also, the squared regression coefficient (*r*_test_^2^) between the observed and predicted values of the test set compounds does not necessarily mean that the predicted values are very near to the observed activities (as there may be considerable numerical difference between the values though maintaining an overall good inter-correlation). To better evaluate the external predictive capacity of a model a modified *r*^2^ term (*r*_m_^2^) is been defined as follows [37]:

(1) $$r_{m}^{2}=r_{test}^{2}\times (1-\sqrt{r_{test}^{2}-r_{o}^{2}})$$

In case of good external prediction capacity, predicted values will be very close to the actual values and thus the *r*_test_^2^ value will be very near to the *r*_0_^2^. In the best case *r*_m_^2^ may be equal to *r*_test_^2^, whereas in the worst case *r*_m_^2^ value could be zero. Herein, the *r*_m_^2^ values of both the two models are larger than the recommended value (0.5). We also noticed that, by comparing the CoMFA with CoMSIA model, the former produces higher predictive power than the latter in terms of both the internal and external exams (Tables 1 and 2), suggesting that the currently developed CoMFA model achieves the predictive task for the novel synthesized compounds better.

### 2.3. Contour Maps

**Figure 3:**
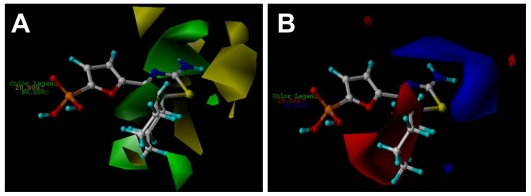
CoMFA StDev*Coeff contour plots with the combination of compound **27**. (**A**) Steric contour map. Green contours indicate regions where bulky groups increase activity (favored level 80%); yellow contours indicate regions where bulky groups decrease activity (disfavored level 20%). (**B**) Electrostatic contour map. Red contours indicate regions where negative charges increase activity (disfavored level 20%); blue contours indicate regions where positive charges increase activity (favored level 80%).

**Figure 4:**
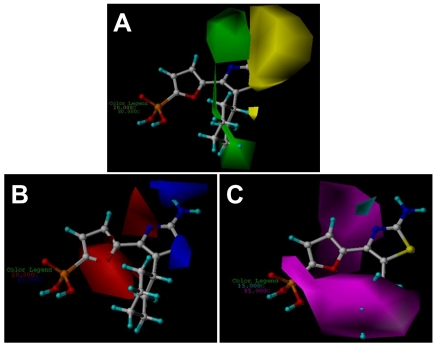
CoMSIA StDev*Coeff contour plots with the combination of compound **27**. (**A**) Steric contour map. Green contours indicate regions where bulky groups increase activity (favored level 80%); yellow contours indicate regions where bulky groups decrease activity (disfavored level 20%). (**B**) Electrostatic contour map. Red contours indicate regions where negative charges increase activity (disfavored level 20%); blue contours indicate regions where positive charges increase activity (favored level 80%). (**C**) H-bond acceptor contour map. Magenta contours indicate regions where H-bond acceptor substituents increase activity (favored level 85%); cyan contours indicate the disfavor regions for H-bond acceptor groups (disfavored level 15%).

The CoMFA and CoMSIA results are represented as 3D coefficient contour maps which show regions where variations of different fields in the structural features of the molecules lead to the increase or decrease of the activity. In this study the most potent compound **27** is exhibited as a representative molecule in the following CoMFA and CoMSIA contour maps (Figures 3 and 4).

The steric and electrostatic fields from the best CoMFA model are represented in Figure 3. In the steric field (Figure 3A), the green-colored contours represent the regions of favorable steric effect, while yellow-colored contours represent regions of unfavorable steric effect, respectively.

As shown in Figure 3A, there exists a large green contour surrounding the cyclohexyl ring linked to the C5 position of the thiazole ring, indicating that the presence of a bulky group in this position will induce an increase of the inhibition activity for the class of compounds. The observation is fully supported by the experimental results. For example, a comparison among C5-thiazole analogues with various alkyl groups (molecules 18–27 shown in Supporting Information, Table S4) comes to a conclusion that the large substituent at 5-position on the thiazole core keeps optimal, since in this position the cyclohexyl ring falls into the green-colored zone. In addition, a careful inspection shows that a small yellow-colored map is located at the distal of the cyclohexyl ring, suggesting that too large groups are disfavored in this position. Taking compound **30**, for example, insertion of a methylene group between the cyclohexyl ring and the thiazole core leads to a decrease of the activity, compared with its counterpart compound **27**. By analyzing the docking simulation as discussed previously, it can also be found that the distal carbon atom of the cyclohexyl core is near the side chain of Asp178 (with a distance of 2.8 Å in the binding pocket). Thus, one conclusion can be drawn that a proper size alkyl group is required at the C5 position to increase the inhibitory activity.

Another large green-colored map situates at C2 position of the thiazole core, indicating the favor of a large substituent group for enhancing the inhibitory potency. However, at the back of this green-colored contour, a yellow-colored map is also observed, suggesting the careful selection of groups of proper size in this position. This investigation is also consistent with the experimental investigation, where for instance, compounds **2** and **3** with larger C2-groups such as ethyl and vinyl groups result in loss of potency compared to their counterpart, compound **1** having a methyl group at the C2 position of the thiazole core (Table S4). We also notice that if both the aryl and heteroaryl groups are introduced to the C2 position, the potency largely decreases that it is much lower (see compounds **15**–**17**) than that of the molecule 1. In addition, an elimination of the C2-Me of compound **1** also leads to a 5-fold weaker compound in thiazole derivative 5, indicating that a suitable C2-group is important for the activity. Our previous investigation of the docking results also finds that the amino group attached to the C2 position of thiazole core forms two hydrogen bonds respectively with the side chain of Thr31 (with a bond length of 1.9 Å) and with the main chain of Val17 (with a bond length of 1.9 Å), which indicates that any larger substitute group might lead to a collision with these residues in the binding pocket, and thus the larger group here is forbidden.

The CoMFA electrostatic contour map with the most potent compound **27** is shown in Figure 3B. Blue contour maps mean that positive-charged substituent groups are beneficial for the activity while red contours indicate that negative charges are conducive. As shown in Figure 3B, blue contour maps are observed surrounding the amino group at the C2 position of the thiazole core suggesting that a charge withdrawing group near these positions enhances the biological activity. The investigation is in agreement with previous docking results. As can be seen in Figure 1, the –NH^2^ group, as a hydrogen bond donor, can interaction with both Thr31 and Val17. In addition, one can also notice that substituent of the hydrogen atom with methyl group (compound **11**) leads to a large loss of potency compared to compound **10** probably due to the lack of the hydrogen bond donor atom. In addition, a large red contour map exists around the phosphate group and embeds the cyclohexyl group illustrating that in these positions electro-rich groups are beneficial for increasing the activity. This may be a reason that all compounds of the dataset contain such electronegative groups (phosphate group).

The CoMSIA steric and electrostatic contour maps (Figure 4A,B) are similar to the CoMFA model and thus are not discussed here additionally. Figure 4C depicts the H-bond acceptor contour map of the CoMSIA model. Magenta contours encompass areas where an H-bond acceptor leads to improved biological activity, while an acceptor located near the cyan regions results in the loss of biological activity. Clearly, it is easily found that a large magenta-colored map is surrounding the phosphate group and the cyclohexyl group, indicating the favor of the presence of H-bond acceptor groups in this region for the activity. The investigation is also supported by previous CoMFA and CoMSIA electrostatic contour maps where, in these positions, a large red map exists, indicating electro-rich groups as hydrogen bond acceptors are beneficial to increase the potency of these inhibitors. Analysis of docking results also shows that the oxygen atoms of the phosphate group as hydrogen acceptors form six hydrogen bonds with the FBPase: two with the side chain hydroxyl groups of Thr27 and Tyr113 with the bond lengths of 2.3 and 1.8 Å respectively; three with the main chain amino groups of Thr27, Glu29 and Lue30 with H-bond lengths of 1.9, 2.7and 1.9 Å, separately; and one with the side chain amino group of Lys112 with the bond length of 1.9 Å. The investigation also consists with the previous report [31]. In addition, a small cyan contour map is near the amino group at C2 position of the thiazole core, indicating the disfavored regions for H-bond acceptor groups. In fact, in the position, hydrogen bond donor groups are favored as depicted in CoMFA and CoMSIA electrostatic contour maps as well as the docking results. Thus this information obtained from the CoMFA and CoMSIA contour maps is helpful to understand the interactions between the inhibitors and the FBPase.

Since the previous report [28] is the subset of the current research, it is very interesting to investigate both the similarity and difference between them. Firstly, we compare the external validation abilities of the best CoMFA models for Chen’s [28] and ours. The best CoMFA model developed by Chen et al. gives *r*_pred_^2^ and *r*_m_^2^ of 0.805 and 0.887, respectively, while, herein the present model exhibits the *r*_pred_^2^ of 0.902, and *r*_m_^2^ of 0.828. Apparently, both the discussed models present the comparable prediction performance. However, it should be noted that the former model was built based on the 63 compounds, while the latter was developed based on 105 molecules with the high statistical confidence. In addition, similar conclusions for modes of interaction between FBPase and inhibitors based the 3D-QSAR models and docking experiments from Chen’s and ours are drawn depicted as follows: (1) At C5 position of the thiazole core, a substituent group with a proper length and size are beneficial to enhance the potency. (2) At C2 position in the thiazole ring, a small and electron-withdraw group is likely to helpful to increase the FBPase inhibition. (3) Substituent groups (such as phosphate) as hydrogen bond acceptors at the C2 position of the furan ring are favored. (4) Several important amino acid residues (such as Thr27, Glu29, Lys112, *etc*.) are identified to play a central role in the ligand binding. In summary, the findings made by Chen complement our findings and offer a clue to designing novel FBPase inhibitors.

### 2.4. MD Simulations

**Figure 5:**
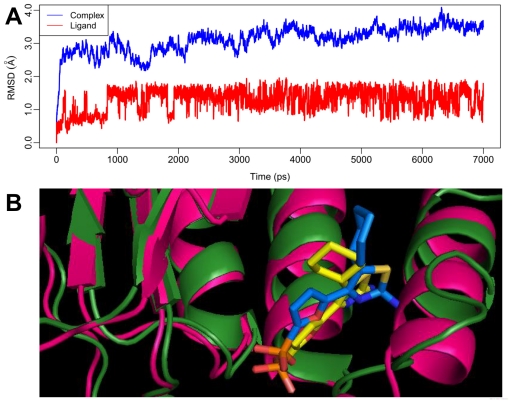
(**A**) Plot of the root-mean-square deviation (RMSD) of docked complex/ligand versus the MD simulation time in the MD-simulated structures. (**B**) View of superimposed backbone atoms of the average structure of the last 1000 ps of the MD simulation (forest green) and the initial structure (hot pink) for inhibitor 27-FBPase complex. Compound **27** is represented in yellow for the initial structure and blue for the final average complex.

In the current investigation, a 7 ns molecular dynamics simulation of the docked complex of FBPase with inhibitor 27 was performed to obtain a dynamic picture of the conformational changes that occur in an aqueous solution, with main emphasis to explore the conformational change that takes place in the inhibitor and the enzyme (Figure 5).

Figure 5A shows the RMSD of the trajectory for the complex with respect to the initial structure (in blue line), the graph presents that the RMSD reaches about 3.5 Å which suggests that a relatively stable conformation of the protein is achieved through the MD simulation. Figure 5A also gives the RMSD of the ligand 27 (in red line) in the binding site of FBPase. It can be noted that the RMSD for the ligand reaches about 1.5 Å after 1 ns of MD simulation and retains this value throughout the simulation.

In order to validate the reliability of the docking results, we have compared the results between the MD and docking simulations in terms of H-bond interactions and van der Waals contacts. By and large, the interaction modes produced between MD and docking simulations share the common features. For example, the phosphate of compound **27** forms three common H-bonds with residues of Thr27, Lys112 and Tyr113. The amino group attached to the thiazole ring forms one common hydrogen bond with Val17. As for van der Waals interactions, both modes basically keep similar interactions. Furthermore, it can also be noticed that there exists slight differences among docking and MD simulation. For the MD results, the phosphate group presents two another H-bonds with residue of Arg140, and lacks the two H-bonds with residues of Glu29 and Leu30 that can be found in the docking results. In addition, the loss of the hydrogen between the amino group attached to thiazole and the residue of Thr31 presented in the docking is compensated by the newly formed H-bond between –NH_2_ with residues of Met18, Glu20 and Arg25 during the MD simulation.

In order to compare the structures from MD simulations and docking, a superimposition of both the structures in the last 1 ns is shown in Figure 5B, where the hotpink ribbon represents the initial structure for the docked complex, the forestgreen ribbon represents the average structure of the MD simulations, with compound **27** represented as stick and line for the initial complex and the average complex, respectively. It can be noticed that, from this figure, there is no significant difference between the average structure extracted from MD simulations and the docked model of the complex, except the cyclohexyl ring exhibiting 90° rotation. However, although the complex has undergone slight movements during MD simulation, both the binding pocket and the conformation of the ligand are still stable, suggesting the rationality and validity of the docking model.

## 3. Materials and Experimental Methods

### 3.1. Dataset

A large, diverse dataset of 105 inhibitors of FBPase were collected from literatures [38,39] published by Dang and co-workers. Here, the converted molar pIC_50_ (−log IC_50_) values, ranging from 4.870 to 8.000 M, were used as the dependent variables in the QSAR regression analysis to improve the normal distribution of the experimental data points. The whole data set was divided into training (77 molecules) and test (28 molecules) sets, respectively. All structures and the corresponding activity values of the dataset as well as their belongings to the training or test set are listed in Table S4 (Supporting Information). Herein, the principle for selection of the test set chemicals was to ensure that, on one hand, their pIC_50_ values are randomly but uniformly distributed in the range of the values for the whole set, and on the other hand, their structures cover as large diversity as possible of the dataset so that the derived models could represent the real characteristics of all the compounds both from the biological activity and the structures.

### 3.2. Molecular Modeling and Alignment

The 3D structures of all compounds were constructed by using the sketch molecule modules of SYBYL6.9 package. Partial atomic charges were calculated by Gasteiger-Hückel method, and energy minimizations were performed by using the Tripos force field and the Powell conjugate gradient algorithm with a convergence criterion of 0.005 kcal/mol.

**Figure 6:**
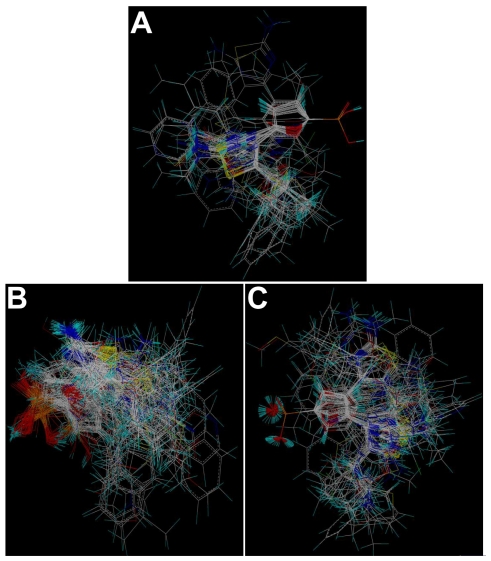
The alignment of all molecules in the dataset. (**A**) Database alignment (Alignment I). (**B**) Alignment from the direct molecular docking conformations (Alignment II). (**C**) Alignment from the combination of both alignments I and II, which means that the molecular active conformations are obtained from molecular docking, while using the same alignment method as that of alignment I (Alignment III).

Molecular alignment of compounds is a crucial step for the successful development of 3D-QSAR models [40]. Thus, in the present work, three alignment methods were performed. Alignment I: In this process, the most potent compound **27** was chosen as a template to fit the remaining training and test set of compounds. Thereafter, all compounds finally minimized with the lowest energy in the dataset were aligned to a common substructure (using the phosphate radical) by substructure-based alignment method using the “align database” command in SYBYL (Figure 6A). Alignment II: It is the alignment from the direct molecular docking conformations (Figure 6B). Alignment III: It is the combination of both alignments I and II, which means that the molecular active conformations are obtained from molecular docking, while using the same alignment method as that of alignment I. Figure 6C presents this alignment result.

### 3.3. Docking Simulation

Docking simulations of thiazoles and oxazoles analogs into the FBPase binding pocket were performed using the Surflex-dock module (V 2.51) of another advanced version of SYBYL package (X 1.1) in this study [41]. The docking method aligns the ligand to a “protomol” or idealized ligand in the active site of the target. The Surflex-dock algorithm and scoring functions have been reported in detail previously [42]. For our studies, the X-ray crystal structure of FBPase with high resolution (2.30 Å) was retrieved from RCSB Protein Data Bank (PDB entry code: 1FTA Chain A). Prior to docking, the ligand and other sub-structures were extracted from the crystal structure, and hydrogen atoms were added to the protein in standard geometry using the biopolymer modulators. In this study, Ligand-based Mode was adopted to generate the protomol in Surflex-dock program. Two adjustable parameters that affect the size and extend of the generated protomol are the threshold and the bloat values. In the present work, the threshold and bloat values were set to 0.5 and 0, respectively. Other parameters were adopted by default values in the Surflex-dock. In the current work, the maximum number of poses per ligand was set to ten. The conformations with the highest total scores for each ligand of the data set were aligned automatically together inside the binding pocket of FBPase and used directly for CoMFA and CoMSIA research.

### 3.4. CoMFA and CoMSIA Interaction Energy Calculations

The steric and electrostatic field energies were calculated using a sp^3^ probe atom with a charge of +1.0 at all intersections of a regularly spaced grid of 2.0 Å in all three dimensions with the defined region. The van der Waals and Coulomb-type potentials representing the steric and electrostatic fields, respectively, were calculated using the Tripos force fields. The grid box dimensions were determined by the created automatically features in the CoMFA module within the SYBYL program. The steric and electrostatic energy values were both truncated to 30.0 kcal/mol.

In CoMSIA, the steric, electrostatic, hydrophobic, hydrogen bond donor and hydrogen bond acceptor potential fields were also calculated at each lattice intersection of a regularly spaced grid of 2.0 Å as that used in CoMFA. A probe atom with radius 1.0 Å and a charge of +1.0 with hydrophobicity of +1.0 and dydrogen bond donor and acceptor properties of +1.0 were used to estimate the steric, electrostatic, hydrophobic, donor and acceptor fields. The attenuation factor α was set to 0.3. CoMSIA similarity indices (*A*_F_) for a molecule *j* with atom *i* at a grid point *q* were calculated by equation (2):

(2) $$A_{F,k}^{q}(j)=-\sum \omega_{probe,k}\omega_{ik}e^{-\alpha \gamma_{iq}^{2}}$$

where *k* represents the steric, electrostatic, hydrophobic, or hydrogen-bond donor or acceptor descriptor. *ω_probe,k_* is the probe atom with radius 1.0 Å, charge +1.0, hydrophobicity +1.0, H-bond donating +1.0, H-bond accepting +1.0; *ω_ik_* is the actual value of the physicochemical property *k* of atom *i; r_iq_* is the mutual distance between the probe atom at grid point *q* and atom *i* of the test molecule.

### 3.5. Partial Lleast Square (PLS) Analysis and Statistical Validation

In the current study, the CoMFA and CoMSIA descriptors served as independent variables and the active values (pIC_50_) as dependent variables in PLS regression analysis for building the 3D-QSAR models. The predictive values of the models were evaluated first by leave-one-out (*LOO*) cross-validation process [43,44]. The cross-validated coefficient, *q*^2^, was calculated using equation (3):

(3) $$q^{2}=1-\frac{\sum_{i=1}^{train}{\left(y_{i}-{\hat{y}}_{i}\right)}^{2}}{\sum_{i=1}^{train}{\left(y_{i}-{\bar{y}}_{tr}\right)}^{2}}$$

where *y_i_*, *ŷ_i_*, and *ȳ_tr_* are the observed, predicted, and mean values of the target property (pIC_50_), respectively for the training set. Herein, the term, $\sum_{i=1}^{train}{\left(y_{i}-{\hat{y}}_{i}\right)}^{2}$, is the predictive residual sum of squares (*PRESS*). The optimal number of components obtained from the cross-validation was used to derive the final QSAR model. Then, a non-cross-validation analysis was carried out; and the Pearson coefficient (*r*_ncv_^2^), standard error of estimates (*SEE*) and the *F* values were calculated. Finally, the CoMFA and CoMSIA results were graphically represented by field contour maps, where the coefficients were generated using the field type “Stdev*Coeff”.

As been reported [32], although the low value of *q*^2^ for the training set can exhibit a low predictive ability of a model, the opposite is not necessarily true. That is, the high *q*^2^ is necessary, but not sufficient, for a model with the high predictive power. Therefore, the external validation must be estimated to establish a reliable and predictive QSAR model. Several often used statistical criteria are listed as follows:

(4) $$r_{pred}^{2}>0.5$$

(5) $$r_{test}^{2}>0.6$$

(6) $$\frac{\left(r_{test}^{2}-r_{o}^{2}\right)}{r_{test}^{2}}<0.1$$

(7) 0.85≤k≤1.15

In equation (4), the predictive *r*^2^, *r*_pred_^2^ is defined as follow:

(8) $$r_{pred}^{2}=1-("PRESS"/SD)$$

where *SD* is the sum of the squared deviations between the actual activity of the compounds in the test set and the mean activity in the training set, and “*PRESS*” is the sum of the squared deviations between predicted and observed activity for each compound in the test set. In equation (4), *r*_test_^2^ is the conventional correlation coefficient between experimental values and model predictions in the test set. The *r*_0_^2^ is a quantity characterizing linear regression with the *Y*-intercept set to zero (*i.e.*, described by *Y* = *kX*, where *Y* and *X* are the actual and predictive activity, respectively). The *k* in equation (6) is the slope of regression lines (predicted versus observed activities) through the origin. The definitions of the afore-mentioned statistical indices are reported in detail in references [32–35].

### 3.6. Molecular Dynamics Simulations

To identify a functionally validated complex from protein docking and the most potent molecule 27, we performed 5 ns molecular dynamics simulations to investigate the conformational changes in the complex induced by the ligand 27. The software AMBER 11 [45] was used for the MD simulations. The inhibitors were minimized using the HF/6-31G* optimization in Gaussian03 [46], and the atom partial charges were obtained by fitting the electrostatic potentials derived by Gaussian via the RESP fitting technique in AMBER 11. The force field parameters for these molecules were assigned by the Antechamber program [47] in AMBER 11. Hydrogen atoms were added to the protein with Tleap module from AMBER. The system was then put in to a rectangular box of TIP3P water molecules [48], and this solvated system contained approximate 59,365 atoms.

The whole systems were minimized in three stages to remove bad contacts between the complex and the solvent molecules. Firstly, the water molecules were minimized by restraining the protein; Secondly, water and the side chains of the protein were minimized by restraining the backbone of the protein, and each stage was performed by using the steepest descent minimization of 2500 steps followed by a conjugate gradient minimization of 2500 steps. Thirdly, the entire system was minimized without any restriction by 10,000 steps changing the minimization method from steepest descent to conjugate gradient after 5000 cycles. After 15,000 steps minimization and equilibration for 60 ps, the system was then heated gradually from 0 to 310 K in the NVT ensemble and equilibrated at 310 K for another 60 ps. After the minimization and heating, 5 ns MD simulations were performed at a constant temperature of 310 K and a constant pressure of 1 atm. The Particle-Mesh Ewald (PME) method [49] was employed to deal with the long-range electrostatic interactions [50] in a periodic boundary condition. The SHAKE algorithm [51] was applied to fix all bond lengths involving hydrogen bonds, permitting a 2-fs time step.

## 4. Conclusions

Presently, a large dataset of 105 thiazoles and oxazoles derivatives as potent orally bioavailable FBPase inhibitors for lowing glucose in type 2 diabetes mellitus has been estimated for the purpose of developing 3D-QSAR models based on both the ligand- and receptor-based superimpositions. Statistically significant models have been derived with two 3D-QSAR methods of CoMFA and CoMSIA on the basis of the database alignment method. The CoMFA model presents higher predictivity than CoMSIA expressed in terms of several rigorous evaluation criteria such as *q*^2^, *r*_pred_^2^ and *r*_m_^2^ for both the internal and external data sets. In addition, both of the two methods pass the *y*-randomization check, suggesting the robustness of the built models. Graphical interpretation of the optimal results, provided by the CoMFA and CoMSIA analyses, brings to light the important structural features that could be responsible for the activity of FBPase inhibitors. (i) Substituents with a proper length and size at the C5 position of the thiazole core are required to enhance the potency; (ii) A small and electron-withdraw group at the C2 position linked to the thiazole core is likely to help to increase the FBPase inhibition; (iii) Substituent groups as hydrogen bond acceptors at the C2 position of the furan ring are favored; (iv) Furthermore, the key amino residues have been found, *i.e.*, Leu30, Glu29, Lys113, Lys112 and Thr27 which form the important hydrogen bond network with the phosphate group of FBPase, and Thr31, Val17 also play an important role in the binding between the ligand and the target.

In addition, a good consistency between the CoMFA and CoMSIA contour maps, molecular docking and molecular dynamics simulations proves the reliability and robustness of the developed models.

Overall, in this report, several reliable computation models between thiazole/oxazole analogues and FBPase have been built, which not only exhibit satisfied statistics, but also provide several possible mechanism interpretations from a molecular-level. We hope the models may provide some instructions for further synthesis of highly potent FBPase inhibitors.

## Supplementary Material



## Figures and Tables

**Table 1:** The optimal comparative molecular field analysis (CoMFA) and comparative molecular similarity indices analysis (CoMSIA) results based on different superimposition methods.

PLS analysis	Superimposition Methods					

	I		II		III	

	CoMFA	CoMSIA	CoMFA	CoMSIA	CoMFA	CoMSIA
*q*^2^	0.514	0.443	0.047	0.191	0.121	0.147
*PCs*	10	6	2	2	4	2
*r*_ncv_^2^	0.986	0.874	0.486	0.485	0.796	0.540
*SEE*	0.108	0.314	0.617	0.618	0.394	0.584
*F* value	462.072	80.809	35.010	34.859	70.348	43.425
*r*_bs_^2^	0.992	0.905	0.635	0.601	0.875	0.630
*SEE*_bs_	0.082	0.267	0.520	0.544	0.304	0.519
*r*_pred_^2^	0.902	0.756	0.364	0.559	0.352	0.473
Relative Contribution (%)						
S	0.563	0.379	0.398	-	0.581	-
E	0.437	0.453	0.602	0.479	0.419	-
H	-	-	-	-	-	-
D	-	-	-	0.521	-	0.822
A	-	0.168	-	-	-	0.178

*q*^2^, cross-validated correlation coefficient after the leave-one-out procedure; *PCs*, principal components; *r*_ncv_^2^, non-cross-validated correlation coefficient; *SEE*, standard error of estimate; *F*, the value of *F* statistic; *r*_bs_^2^, the average *r*^2^ value from a bootstrapping analysis for 100 runs; *SEE*_bs_, the average *SEE* value from a bootstrapping analysis for 100 runs; *r*_pred_^2^, predicted correlation coefficient for the test set of compounds. Superimposition method: I, from the database alignment; II, from docking alignment; III, from database alignment based on the docking conformations.

**Table 2:** Comparison of the external predictability of the optimal CoMFA and CoMSIA, for the prediction set

**Model**	*r*_test_^2^	*r*_pred_^2^	*r*_0_^2^	(*r*_test_^2^ − *r*_0_^2^)/*r*_test_^2^	*r*^2^_m_	*k*	*k*′
**CoMFA**	0.909	0.902	0.901	0.009	0.828	0.981	1.018
**CoMSIA**	0.741	0.756	0.693	0.065	0.579	0.990	1.005

*r*_test_^2^, conventional *r*^2^ in the test set; *r*_pred_^2^, predicted correlation coefficient for the test set of compounds; *r*_0_^2^, *r*^2^ with the *Y*-intercept set to zero; *k*, slope of regression lines (observed versus predicted activities) through the origin; *k*′ slope of regression lines (predicted versus observed activities) through the origin.
