# Supplementary material for: In Silico Identification of Structure Requirement for Novel Thiazole and Oxazole Derivatives as Potent Fructose 1,6-Bisphosphatase Inhibitors
Source: Int J Mol Sci. 2011 Nov 18;12(11):8161–80. doi: 10.3390/ijms12118161 (PMC3233463; doi:10.3390/ijms12118161)
Supplement: Supplementary file 1 [file ijms-12-08161-s001.pdf]

Supplementary Information

Ming Hao [1,†], Xiaole Zhang [2,†], Hong Ren [3,4], Yan Li [1,*], Shuwei Zhang [1], Fang Luo [5], Mingjuan Ji [5], Guohui Li [4] and Ling Yang [6]

1 Department of Materials Science and Chemical Engineering, Dalian University of Technology, Dalian, Liaoning, 116023, China; E-Mails: dluthm@yeah.net (M.H.); zswei@dlut.edu.cn (S.Z.)

2 Department of Mathematical Sciences, Dalian University of Technology, Dalian, Liaoning, 116023, China; E-Mail: xlfree@foxmail.com

3 Department of Ophthalmology, Qi Lu Hospital, Medical School of Shandong University, Jinan, 250012, China; E-Mail: renhong999@sina.com

4 Laboratory of Molecular Modeling and Design, State Key Laboratory of Molecular Reaction Dynamics, Dalian Institute of Chemical Physics, Chinese Academy of Sciences, Dalian, 116023, China; E-Mail: ghli@dicp.ac.cn

5 College of Chemistry and Chemical Engineering, Graduate School of the Chinese Academy of Sciences, Beijing, 100049, China; E-Mails: luofang09b@mails.gucas.ac.cn (F.L.); jmj@gucas.ac.cn (M.J.)

6 Laboratory of Pharmaceutical Resource Discovery, Dalian Institute of Chemical Physics, Graduate School of the Chinese Academy of Sciences, Dalian, Liaoning, 116023, China; E-Mail: yling@dicp.ac.cn

† These authors contributed equally to this work.

* Author to whom correspondence should be addressed; E-Mail: yanli@dlut.edu.cn; Tel.: +86-411-84986062; Fax: +86-411-84986063.

Received: 8 October 2011; in revised form: 10 November 2011 / Accepted: 16 November 2011 / Published: 18 November 2011

Abstract: Fructose 1,6-bisphosphatase (FBPase) has been identified as a drug discovery target for lowering glucose in type 2 diabetes mellitus. In this study, a large series of 105 FBPase inhibitors were studied using a combinational method by 3D-QSAR, molecular docking and molecular dynamics simulations for a further improvement in potency. The optimal 3D models exhibit high statistical significance of the results, especially for the CoMFA results with r_{ncv}^2, q^2 values of 0.986, 0.514 for internal validation, and r_{pred}^2, r_{m}^2 statistics of 0.902, 0.828 statistics for external validation. Graphic representation of the results, as contoured 3D coefficient plots, also provides a clue to the reasonable modification of molecules. (1) Substituents with a proper length and size at the C5 position of the thiazole core are required to enhance the potency; (2) A small and electron-withdrawing group at the C2 position linked to the thiazole core is likely to help increase the FBPase inhibition; (3) Substituent groups as hydrogen bond acceptors at the C2 position of the furan ring are favored. In addition, the agreement between 3D-QSAR, molecular docking and molecular

dynamics simulation proves the rationality of the developed models. These results, we hope, may be helpful in designing novel and potential FBPase inhibitors.

Keywords: 3D-QSAR; molecular dynamics; FBPase inhibitors; CoMFA; CoMSIA

Table S1. The actual and predicted values (pIC_{50}) based on the optimal CoMFA and CoMSIA models from superimposition-I, II and III.

		Superimposition Methods					
		I		II		III	
Compd.	Actual	CoMFA	CoMSIA	CoMFA	CoMSIA	CoMFA	CoMSIA
1	7.000	6.867	6.892	6.993	6.934	7.114	6.008
2	6.400	6.384	6.588	5.505	5.656	6.163	5.702
3	5.920	6.102	6.060	5.853	5.892	6.063	5.964
4	6.660	6.791	6.403	7.049	7.117	7.139	6.176
5	6.300	6.406	6.998	6.291	6.029	6.520	5.997
6	6.740	6.606	6.684	6.056	6.177	6.329	5.744
7	7.100	6.925	6.729	5.534	5.702	6.267	5.685
8	6.050	6.033	6.569	5.931	6.114	6.367	5.715
9	5.700	5.732	5.703	6.423	6.257	5.673	5.945
10	7.600	7.375	7.058	7.353	7.455	7.631	7.367
11	6.000	5.983	6.633	6.036	5.970	6.184	5.805
12	5.000	5.001	5.146	5.320	5.483	4.763	5.635
13	5.560	5.473	5.661	5.867	6.034	5.643	5.769
14 *	6.300	6.126	6.101	6.022	6.479	6.038	6.052
15	4.870	4.839	4.934	5.261	5.488	4.884	5.809
16 *	5.100	4.714	5.492	6.180	6.491	5.763	5.803
17	5.300	5.35	5.072	6.421	6.118	5.282	5.927
18	6.350	6.154	6.605	6.399	6.746	6.245	6.235
19	6.920	6.994	6.789	6.729	6.682	6.699	7.283
20	6.300	6.308	6.220	6.406	6.304	6.111	6.987
21	7.520	7.603	7.086	7.436	7.476	7.616	7.393
22 *	7.550	7.088	6.980	7.533	7.317	8.029	7.970
23	7.240	7.281	7.127	7.418	7.635	6.886	7.387
24	7.920	7.988	7.747	7.417	7.258	7.900	6.960
25	7.720	7.672	7.695	7.432	6.948	6.987	6.879
26 *	7.680	7.712	7.870	7.259	7.379	7.195	7.158
27	8.000	8.071	7.992	7.432	7.313	7.189	7.908
28 *	7.700	7.558	7.397	7.267	7.431	6.919	7.378
29	7.740	7.77	7.658	6.512	6.297	6.768	6.912
30 *	7.230	7.167	7.680	7.112	6.965	6.964	6.879
31	6.820	6.84	6.706	6.695	6.088	6.935	6.605
32	6.250	6.281	5.788	6.992	6.795	6.517	6.690
33	7.150	7.118	6.698	6.721	6.673	7.581	6.964
34 *	7.300	7.026	6.652	6.657	6.747	5.982	5.667
35	7.000	7.119	6.664	6.816	6.757	6.913	6.822
36 *	7.800	8.099	7.966	7.339	7.401	7.194	7.678
37	7.480	7.666	7.258	6.646	7.054	7.187	7.202
38 *	7.800	7.125	7.024	7.020	6.819	7.117	6.739
39	7.620	7.56	7.622	7.229	7.736	7.060	7.092

Table S1. *Cont.*

40 *	7.620	7.466	6.626	7.452	7.383	7.549	6.943
41	6.520	6.442	6.987	6.450	6.012	6.441	6.696
42	5.770	5.880	6.111	6.843	6.913	5.902	7.404
43	7.850	7.854	8.394	7.226	7.024	7.967	7.746
44 *	7.820	7.600	8.379	7.235	6.923	6.329	6.167
45	6.070	6.044	5.815	6.247	6.277	6.129	6.34
46	7.850	7.869	7.854	6.972	7.633	7.297	7.225
47	7.370	7.375	7.852	6.879	7.090	7.606	7.267
48 *	7.680	7.532	7.857	6.879	7.326	6.476	7.121
49	7.660	7.712	7.691	6.978	6.813	7.325	6.910
50 *	7.680	7.758	7.844	7.303	7.364	7.281	7.409
51	7.060	7.029	6.985	7.138	6.949	6.950	7.051
52 *	7.850	7.579	7.652	7.193	7.382	7.319	7.422
53	7.800	7.847	7.778	7.487	7.394	7.358	7.437
54 *	7.890	7.702	7.768	7.356	7.311	7.825	7.851
55	7.490	7.404	7.524	7.596	7.454	7.768	7.509
56 *	7.390	7.442	7.843	6.967	7.426	7.087	6.597
57	7.470	7.506	7.651	7.260	7.387	7.514	7.323
58 *	7.920	7.740	7.614	7.019	7.175	6.871	7.360
59	7.400	7.410	7.276	7.372	7.214	7.384	7.824
60 *	7.360	7.385	7.612	7.433	7.416	7.350	7.404
61	5.000	4.962	5.261	7.328	7.034	5.815	6.650
62	7.300	7.408	6.777	6.679	7.116	7.215	6.659
63	6.020	5.980	5.783	5.682	5.724	5.613	6.267
64 *	5.700	5.218	5.895	7.539	7.302	7.154	6.277
65	5.890	5.935	5.869	6.223	6.214	6.456	5.855
66	6.870	6.902	6.831	6.420	6.648	7.130	6.414
67	6.680	6.722	6.668	7.216	6.770	7.119	6.568
68	7.100	7.009	6.954	6.624	6.549	6.496	6.464
69 *	6.920	6.279	6.203	6.549	6.337	6.272	6.124
70	5.000	5.411	6.116	6.282	6.466	5.470	5.444
71 *	6.850	6.496	6.165	6.378	6.382	6.825	5.631
72	6.770	6.844	6.713	6.979	7.595	6.977	6.999
73	6.680	6.582	6.826	6.919	6.898	6.713	6.714
74	6.490	6.494	6.969	7.120	7.541	6.809	6.931
75 *	6.800	6.203	6.118	5.635	6.680	6.864	6.640
76	6.050	6.052	5.871	6.245	5.938	6.044	6.289
77	6.590	6.592	6.560	7.175	6.819	6.802	6.847
78	7.150	7.085	6.710	7.086	6.782	7.028	6.917
79	6.960	6.977	6.815	6.830	7.213	6.761	6.962
80	6.920	6.893	6.405	6.161	6.524	6.595	6.809
81 *	5.400	5.716	5.966	7.153	6.822	6.756	6.925
82	7.170	7.172	6.975	6.571	7.035	7.893	6.927
83	7.420	7.253	7.260	6.986	6.874	7.879	7.623
84	7.400	7.375	7.233	6.816	6.809	6.425	7.757
85	7.070	7.088	7.182	7.016	6.489	7.524	7.007
86	7.520	7.520	7.363	6.972	6.510	7.633	6.908

Table S1. *Cont.*

87	6.070	5.953	6.168	5.839	6.190	6.431	6.789
88	6.220	6.047	5.945	5.924	5.887	6.568	6.007
89	5.000	5.037	5.325	5.364	5.535	5.667	5.776
90 *	5.720	6.005	6.309	6.620	6.218	7.361	6.207
91	5.680	5.497	5.263	5.759	5.856	5.820	5.994
92 *	5.660	5.849	5.440	6.198	5.758	5.443	5.828
93	5.370	5.553	5.585	6.314	6.046	5.714	6.101
94 *	6.020	6.085	5.776	6.076	5.672	5.983	5.786
95	5.000	5.092	5.094	5.842	5.515	5.439	6.423
96 *	5.170	5.463	5.458	6.098	5.687	5.680	5.768
97	5.150	5.228	5.443	6.170	6.313	5.644	6.113
98	6.380	6.231	6.163	6.305	6.357	5.992	6.258
99	6.420	6.557	6.489	6.761	6.635	6.577	6.207
100	6.550	6.517	6.557	6.800	6.978	6.311	6.669
101	6.240	6.193	6.431	6.901	6.670	6.294	6.238
102	6.600	6.573	6.677	6.827	6.704	6.237	6.052
103	6.460	6.435	6.635	6.382	6.398	6.413	6.128
104 *	5.000	5.397	6.067	6.675	6.120	6.454	5.490
105	5.000	4.900	5.112	5.591	5.751	4.972	5.662

* test set. Superimposition method: I, from the database alignment; II, from docking alignment; III, from database alignment based on the docking conformations.

Table S2. Comparison the q^2, r_{ncv}^2 and *SEE* values for the first 11 components of the optimal CoMFA model (Superposition I).

	Optimal CoMFA model		
No. of *PCs*	q^2	r_{ncv}^2	*SEE*
1	0.211	0.342	0.694
2	0.222	0.453	0.637
3	0.284	0.727	0.453
4	0.341	0.829	0.361
5	0.434	0.886	0.297
6	0.458	0.934	0.227
7	0458	0.934	0.227
8	0.474	0.968	0.161
9	0.496	0.976	0.139
10	0.514	0.986	0.108
11	0.514	0.986	0.108

Table S3. Comparison the q^2, r_{ncv}^2 and *SEE* values for the first 7 components of the optimal CoMSIA model (Superposition I).

	Optimal CoMSIA model		
No. of *PCs*	q^2	r_{ncv}^2	*SEE*
1	0.154	0.304	0.714
2	0.237	0.555	0.574
3	0.305	0.670	0.498
4	0.362	0.770	0.419
5	0.429	0.828	0.365
6	0.443	0.874	0.314
7	0.443	0.874	0.314

Table S4. Compounds with their chemical names, activities and classes used in the dataset.

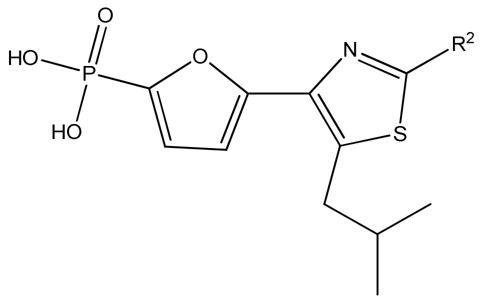

No.	R^2	pIC_{50}	Ref. [a]
1	Me	7.000	[38]
2	Et	6.400	[38]
3	vinyl	5.920	[38]
4	CH_2OH	6.660	[38]
5	H	6.300	[38]
6	Cl	6.740	[38]
7	Br	7.100	[38]
8	SMe	6.050	[38]
9	CN	5.700	[38]
10	NH_2	7.600	[38]
11	NHMe	6.000	[38]
12	NHAc	5.000	[38]
13	$CONH_2$	5.560	[38]
14 *	$CSNH_2$	6.300	[38]
15	Ph	4.870	[38]
16 *	2-thienyl	5.100	[38]
17	3-pyridyl	5.300	[38]

Table S4. *Cont.*

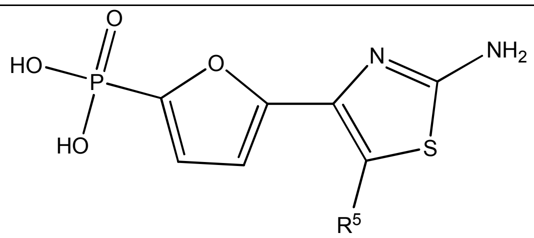

No.	R^5	pIC_{50}	Ref. [a]
18	H	6.350	[38]
19	Me	6.920	[38]
20	$HOCH_2$	6.300	[38]
21	*n*-Pr	7.520	[38]
22 *	*i*-Pr	7.550	[38]
23	CF_3CH_2	7.240	[38]
24	neopentyl	7.920	[38]
25	cyclobutyl	7.720	[38]
26 *	cyclopentyl	7.680	[38]
27	cyclohexyl	8.000	[38]
28 *	cyclopropyl-CH_2	7.700	[38]
29	cyclopentyl-CH_2	7.740	[38]
30 *	cyclohexyl-CH_2	7.230	[38]
31	$PhCH_2$	6.820	[38]
32	morpholinyl-CH_2	6.250	[38]

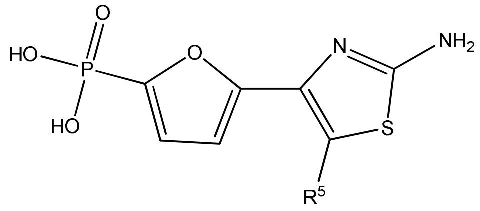

No.	R^5	pIC_{50}	Ref. [a]
33	Cl	7.150	[38]
34 *	Br	7.300	[38]
35	I	7.000	[38]
36 *	1-morpholinyl	7.800	[38]
37	EtS	7.480	[38]
38 *	*n*-PrS	7.800	[38]
39	*i*-PrS	7.620	[38]
40 *	*t*-BuS	7.620	[38]
41	PhS	6.520	[38]
42	$CONMe_2$	5.770	[38]
43	CO_2Et	7.850	[38]
44 *	CO_2Bn	7.820	[38]
45	*n*-PrSO	6.070	[38]
46	Ph	7.850	[38]
47	2-MeO-Ph	7.370	[38]
48 *	3-MeO-Ph	7.680	[38]
49	4-MeO-Ph	7.660	[38]

Table S4. *Cont.*

50 *	4-MeS-Ph	7.680	[38]
51	4-*t*-Bu-Ph	7.060	[38]
52 *	4-MeO_2C-Ph	7.850	[38]
53	4-F-Ph	7.800	[38]
54	4-Cl-Ph	7.890	[38]
55	4-Ac-Ph	7.490	[38]
56 *	4-$MeSO_2$-Ph	7.390	[38]
57	4-Ph-Ph	7.470	[38]
58 *	2-nathphyl	7.920	[38]
59	2-furanyl	7.400	[38]
60 *	2-thienyl	7.360	[38]

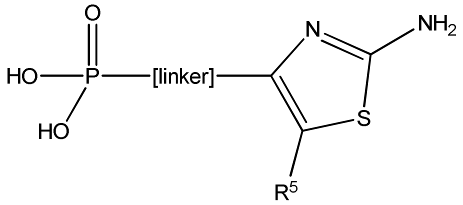

No.	linker	R^5	PIC_{50}	Ref. [a]
61	2,5-furanyl	H	5.00	[38]
62	–CH_2OCO–	*n*-Pr	7.300	[38]
63	–CH_2NHCO–	2-thienyl	6.020	[38]
64 *	2,6-pyridyl	H	5.700	[38]
65	1,3-phenyl	H	5.890	[38]
66	1,3-phenyl-(6-Me)	*n*-Pr	6.870	[38]
67	1,3-phenyl-(6-OMe)	*i*-Pr	6.680	[38]
68	1,3-phenyl-(6-F)	Ph	7.100	[38]

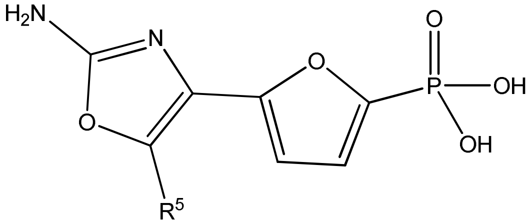

No.	R^5	PIC_{50}	Ref. [a]
69 *	*i*-Bu	6.920	[39]
70	H	5.000	[39]
71 *	Allyl	6.850	[39]
72	*n*-Bu	6.770	[39]
73	*n*-Pentyl	6.680	[39]
74	–CH_2-cyclohexyl	6.490	[39]
75 *	Ph	6.800	[39]
76	Bn	6.050	[39]
77	–CH_2-(2-thienyl)	6.590	[39]
78	*n*-PrS	7.150	[39]
79	*i*-PrS	6.960	[39]
80	*t*-BuS	6.920	[39]
81 *	PhS	5.400	[39]

Table S4. *Cont.*

82	$–CO_2Me$	7.170	[39]
83	$–CO_2Et$	7.420	[39]
84	$–CO_2Pr$-*i*	7.400	[39]
85	$–CO_2Bn$	7.070	[39]
86	–COSEt	7.520	[39]
87	–COBu-*t*	6.070	[39]

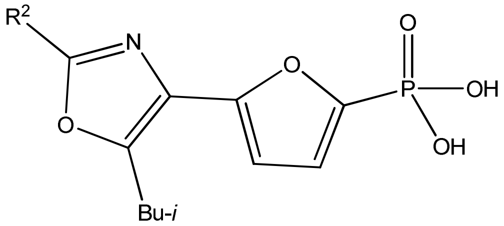

No.	R^2	pIC_{50}	Ref. [a]
88	Me	6.220	[39]
89	HO	5.000	[39]
90 *	H	5.720	[39]
91	Me_2N–	5.680	[39]
92 *	*i*-Pr-	5.660	[39]
93	MeHN–	5.370	[39]
94 *	Et	6.020	[39]
95	EtHN–	5.000	[39]
96 *	vinyl	5.170	[39]

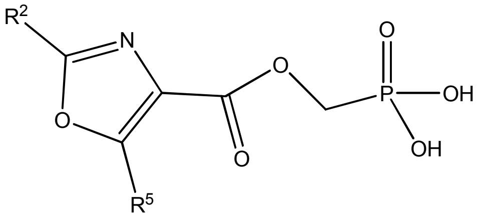

No.	R^2	R^5	Ref. [a]	No.
97	H_2N–	H	5.150	[39]
98	H_2N–	Me	6.380	[39]
99	H_2N–	Et	6.420	[39]
100	H_2N–	*n*-Pr	6.550	[39]
101	H_2N–	*i*-Pr	6.240	[39]
102	H_2N–	*n*-Bu	6.600	[39]
103	H_2N–	*n*-Pent	6.460	[39]
104 *	Me	CF_3	5.000	[39]
105	H	Ph	5.000	[39]

* test set; [a] from the corresponding reference.

© 2011 by the authors; licensee MDPI, Basel, Switzerland. This article is an open access article distributed under the terms and conditions of the Creative Commons Attribution license (http://creativecommons.org/licenses/by/3.0/).
